# Physical activity practiced at a young age is associated with a less severe subsequent clinical presentation in facioscapulohumeral muscular dystrophy

**DOI:** 10.1186/s12891-023-07150-x

**Published:** 2024-01-05

**Authors:** Cinzia Bettio, Federico Banchelli, Valentina Salsi, Roberto Vicini, Oscar Crisafulli, Lucia Ruggiero, Giulia Ricci, Elisabetta Bucci, Corrado Angelini, Angela Berardinelli, Silvia Bonanno, Maria Grazia D’Angelo, Antonio Di Muzio, Massimiliano Filosto, Erica Frezza, Lorenzo Maggi, Tiziana Mongini, Elena Pegoraro, Carmelo Rodolico, Marina Scarlato, Gaetano Vattemi, Daniele Velardo, Giuliano Tomelleri, Roberto D’Amico, Giuseppe D’Antona, Rossella Tupler

**Affiliations:** 1 Department of Biomedical, Metabolic and Neural Sciences, University of Modena and Reggio Emilia, via G. Campi 287, Modena, 41125 Italy; 2 Unit of Statistical and Methodological Support to Clinical Research, Azienda Ospedaliero-Universitaria, Modena, Italy; 3 Department of Life Sciences, University of Modena and Reggio Emilia, Modena, Italy; 4 Centro di Ricerca Interdipartimentale nelle Attività Motorie e Sportive (CRIAMS)-Sport Medicine Centre, University of Pavia, Voghera, Italy; 5 Department of Neurosciences, Reproductive and Odontostomatological Sciences, University Federico II of Naples, Naples, Italy; 6 Department of Clinical and Experimental Medicine, Neurological Clinic, University of Pisa, Pisa, Italy; 7 Department of Neuroscience, Mental Health and Sensory Organs, S. Andrea Hospital, University of Rome “La Sapienza”, Rome, Italy; 8 IRCCS Fondazione San Camillo Hospital, Venice, Italy; 9 Unit of Child Neurology and Psychiatry, IRCCS “C. Mondino” Foundation, Pavia, Italy; 10 Neuroimmunology and Neuromuscular diseases Unit, Fondazione IRCCS Istituto Neurologico Carlo Besta, Milan, Italy; 11 NeuroMuscular Unit, Scientific Institute IRCCS E. Medea, Bosisio Parini (Lecco), Italy; 12 Center for Neuromuscular Disease, CeSI, University “G. D’Annunzio”, Chieti, Italy; 13 Neurology Clinic, University of Brescia, Brescia, Italy; 14 Unit Malattie Neuromuscolari, Policlinico e Università di Roma Tor Vergata, Roma, Italy; 15 Department of Neurosciences “Rita Levi Montalcini”, Center for Neuromuscular Diseases, University of Turin, Turin, Italy; 16 Department of Neurosciences, University of Padua, Padua, Italy; 17 Department of Clinical and Experimental Medicine, University of Messina, Messina, Italy; 18 INSPE and Division of Neuroscience, IRCCS San Raffaele Scientific Institute, Milan, Italy; 19 Department of Neurosciences, Biomedicine and Movement Sciences, Section of Clinical Neurology, University of Verona, Verona, Italy; 20 Neurology Unit, Foundation IRCCS Ca’ Granda Ospedale Maggiore Policlinico, Milan, Italy; 21 Department of Medical and Surgical Sciences, University of Modena and Reggio Emilia, Modena, Italy; 22 Department of Public Health, Experimental and Forensic Medicine, University of Pavia, Pavia, Italy; 23 Department of Molecular Cell and Cancer Biology, University of Massachusetts Medical School, Worcester, USA; 24 Li Weibo Institute for Rare Diseases Research, University of Massachusetts Medical School, Worcester, USA

**Keywords:** FSHD, Neuromuscular diseases, Sport medicine, Physical activity, Rare disease, Health promotion

## Abstract

**Background:**

In facioscapulohumeral muscular dystrophy (FSHD), it is not known whether physical activity (PA) practiced at young age is associated with the clinical presentation of disease. To assess this issue, we performed a retrospective cohort study concerning the previous practice of sports and, among them, those with medium-high cardiovascular commitment in clinically categorized carriers of a D4Z4 reduced allele (DRA).

**Methods:**

People aged between 18 and 60 were recruited as being DRA carriers. Subcategory (classical phenotype, A; incomplete phenotype, B; asymptomatic carriers, C; complex phenotype, D) and FSHD score, which measures muscle functional impairment, were assessed for all participants. Information on PAs was retrieved by using an online survey dealing with the practice of sports at a young age.

**Results:**

368 participants were included in the study, average age 36.6 years (SD = 9.4), 47.6% male. The FSHD subcategory A was observed in 157 (42.7%) participants with average (± SD) FSHD score of 5.8 ± 3.0; the incomplete phenotype (category B) in 46 (12.5%) participants (average score 2.2 ± 1.7) and the D phenotype in 61 (16.6%, average score 6.5 ± 3.8). Asymptomatic carriers were 104 (subcategory C, 28.3%, score 0.0 ± 0.2). Time from symptoms onset was higher for patients with A (15.8 ± 11.1 years) and D phenotype (13.3 ± 11.9) than for patients with B phenotype (7.3 ± 9.0). The practice of sports was associated with lower FSHD score (-17%) in participants with A phenotype (MR = 0.83, 95% CI = 0.73–0.95, *p* = 0.007) and by 33% in participants with D phenotype (MR = 0.67, 95% CI = 0.51–0.89, *p* = 0.006). Conversely, no improvement was observed in participants with incomplete phenotype with mild severity (B).

**Conclusions:**

PAs at a young age are associated with a lower clinical score in the adult A and D FSHD subcategories. These results corroborate the need to consider PAs at the young age as a fundamental indicator for the correct clinical stratification of the disease and its possible evolution.

**Supplementary Information:**

The online version contains supplementary material available at 10.1186/s12891-023-07150-x.

## Background

FSHD is the second most common progressive hereditary muscular dystrophy in adults with an estimated prevalence of 1 in 20,000 individuals [1]. The disease has a wide phenotypic spectrum, including heterogeneous patterns of symptoms and progression [2–5] and a variable age at onset, mainly in the second or third decade of life [6, 7]. To overcome such complexity in the clinical presentation, patients are currently allocated to four clinical categories according to the Comprehensive Clinical Evaluation Form (CCEF) [8], which classifies (1) subjects with facial and scapular girdle muscle weakness (category A), (2) subjects with muscle weakness limited to the scapular girdle or facial muscles (category B), (3) asymptomatic/healthy subjects (category C), and (4) subjects with a myopathic phenotype presenting clinical features not consistent with the canonical phenotype of FSHD (category D). Overall, patients belonging to category A have the most peculiar signs of the disease. The effectiveness of physical activities (PAs), term which includes both daily life and structured activities (exercise), whether recreational or competitive [9], in FSHD is still debated [10]. In 2019, a Cochrane review highlighted that aerobic exercise training may have positive effects by leading to an improvement of the aerobic fitness [11], but it is currently unknown whether having practiced PAs at the young age may have an impact on the clinical presentation and, if so, whether this is positive or negative and if it varies across the clinical categories. Unraveling this open question is relevant, especially for patients and their relatives. In our experience, people who come for a genetic consultation or a neurological examination ask about the safety of playing sports or having been involved in PAs. Unfortunately, pressing questions such as: “I am a carrier of a neuromuscular disease (NMD) genetic defect: can I play sports safely?” or “I suffer from an NMD, what kind of sport can I play?” or else “Is playing sports detrimental for my condition?” or “Did my past involvement in sports accelerate the disease progression and severity?” still need clear answers. In healthy subjects, an increasing amount of evidence suggests that PAs practiced in young age have a crucial role in maintaining a good state of physical health and efficiency in the adulthood [12, 13]. A systematic review with meta-analysis of longitudinal studies, including a total of 21.686 participants [14], points out that a high level of physical fitness in childhood and adolescence is associated with several positive effects in subsequent stages of life such as lower body mass index (BMI) and skinfold thickness, reduced insulin resistance, lower cardiovascular disease risk score, amelioration of the lipid profile, and higher bone mineral density. In this light, addressing the possible association between PAs performed at young age and the clinical presentation of FSHD could be of major importance to give evidence-based indications to D4Z4 reduced allele (DRA) carriers. Furthermore, verification of the possible differences in the effects of previous PAs in the various clinical subcategories could constitute a further factor in the stratification of these patients. To investigate these aspects, taking advantage of the subjects accrued by the Italian National Registry for FSHD (INRF), which gather data from molecular analysis, clinical evaluation, anamnestic information, and family history [15], we retrospectively investigated the association between PAs previously practiced in the range of age between 6 and 30, and the disease expression in 368 DRA carriers.

## Methods

### Aims

The primary aim of the study was to assess the association between PAs practiced between 6 and 30 years of age and the FSHD score collected at the age of the first clinical evaluation in DRA carriers clinically subdivided in classical phenotype (category A), incomplete phenotype (category B), asymptomatic carriers (category C), and atypical phenotype (category D) according to the Comprehensive Clinical Evaluation Form (CCEF) [8]. The secondary aim of the study was to assess whether the FSHD clinical score of the participants correlates with the practice of PAs in organized settings, which refer to the use of sports halls or fields with the availability of appropriate equipment for sports activities (team or individual) [16] and/or with PAs classified based on the cardiovascular commitment.

### Study design

This study followed a retrospective cohort study design and was carried out in accordance with the STROBE statement [17].

### Selection of participants

**Fig. 1:**
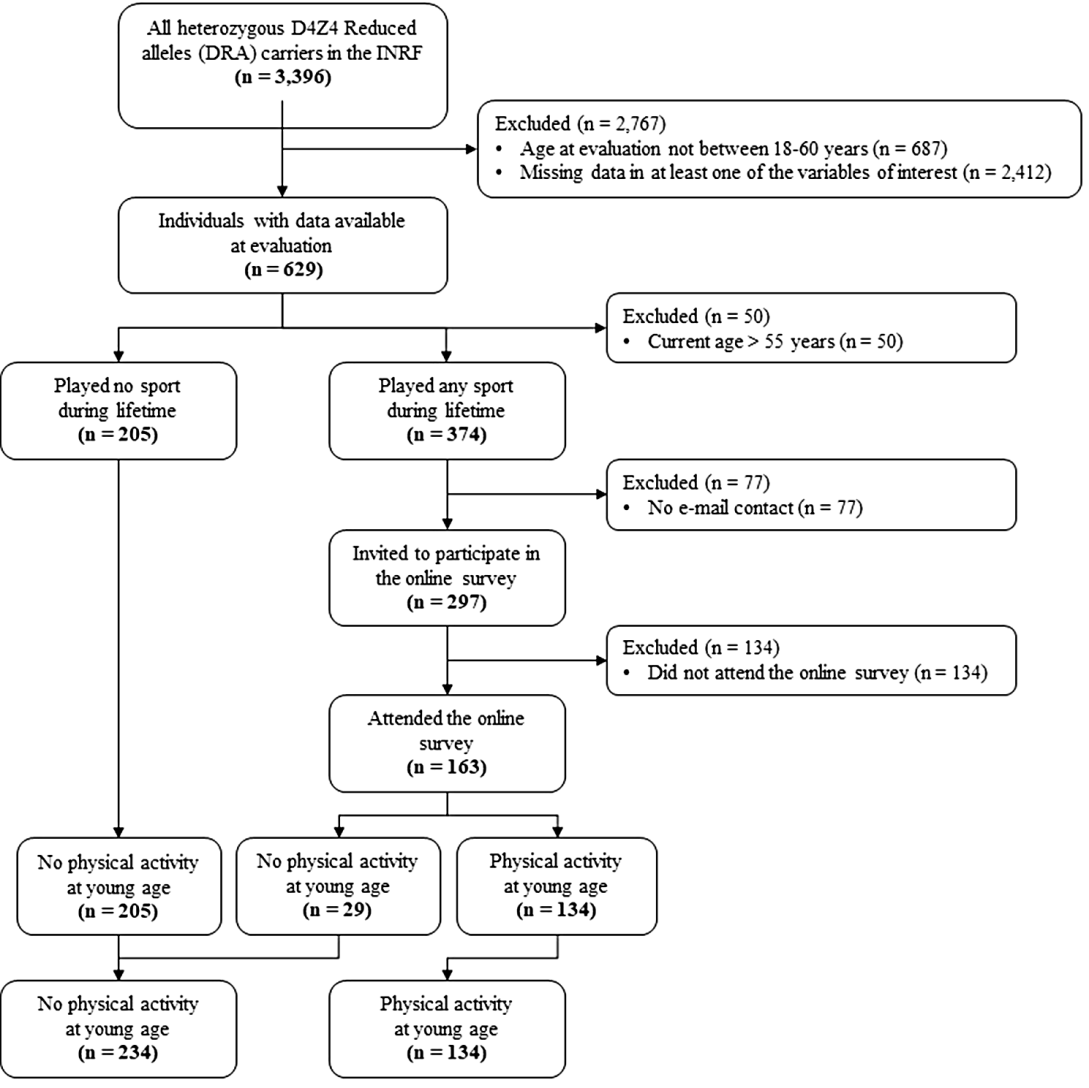
Flowchart of enrollment and selection of subjects for participating in the online survey

Inclusion criteria for the study were: being a DRA carrier registered in the INRF; age at FSHD score evaluation between 18 and 60 years. The only exclusion criterion was to have missing values in at least one of the variables of interest (gender, FSHD score, age at FSHD score assessment, age at symptoms onset, FSHD subcategory, DRA size, PAs practice). Participants who were eligible for inclusion were divided into two groups: those who had practiced PAs in their lifetime and those who had not. The latter were included in the analysis, whereas the formers were invited to participate in an online survey about the characteristics of PAs practiced between 6 and 30 years of age, which represent the period when most people practice PAs [18]. Participants who attended the online survey were included in the analysis. Given the retrospective nature of the study no formal calculation of sample size was carried out. The flowchart of enrollment and selection of subjects for participating in the online survey is described in Fig. 1.

### Classification of physical activities

Only PAs practiced at least twice a week and for a consecutive period longer than three months were considered. For participants aged less than 30 years at clinical evaluation, only PAs that were practiced before that age were considered.

Organized PAs were defined as those that were organized in training sessions held regularly with the use of sports halls or fields and with the availability of appropriate equipment (team or individual) [16]. PAs were classified in groups A, B, C, and D1 and D2 based on the cardiovascular commitment according to the guidelines for competitive sports issued by the Italian Society of Sports Cardiology [19]. Sport disciplines were grouped as follows: Group A, little-to-moderate change in Heart Rate (HR) and Cardiac Output (CO); Group B, moderate-to-high increase in HR and minimal change in CO and Peripheral Resistance (PR); Group C, moderate-to-high increase in HR and PR and submaximal CO; Group D1 mixed moderate-to-high cardiovascular commitment and group D2, submaximal or maximal increase in HR and CO and reduced PR. For our purposes A and B groups were identified as sport disciplines with Low Cardiovascular Commitment (LCC), group C as sport disciplines with Moderate Cardiovascular Commitment (MCC), and D1 and D2 as sports with Moderate-High Cardiovascular Commitment (MHCC). Classification criteria regarding changes in HR, PR and CO in groups A, B, C, D1 and D2 are synoptically summarized in supplemental Table 1. Sports classification related to the enrolled participants is described in supplemental Table 2.

### Clinical evaluation

The CCEF [8] was used to classify all DRA carriers. The CCEF divides the carriers into 4 classes of phenotype, as follows: (1) individuals presenting facial and scapular girdle muscle weakness typical of FSHD (category A, subcategories A1–A3), (2) individuals with muscle weakness limited to scapular girdle or facial muscles (category B subcategories B1, B2), (3) asymptomatic/healthy individuals (category C, subcategories C1, C2), (4) individuals with myopathic phenotype presenting clinical features not consistent with FSHD canonical phenotype (D, subcategories D1, D2). The CCEF also assigned a numerical value to the muscle impairment, the FSHD score, of six different muscular groups typically involved in FSHD: face muscles (score 0–2); shoulder girdle muscles (score 0–3); upper limbs muscles (score 0–2); distal legs muscles (score 0–2); pelvic girdle muscles (score 0–5); abdominal muscles (score 0–1). The sum of these scores ranges from 0, when no objective sign of functional impairment is present, to 15, when all tested muscle groups are severely impaired, and the patient is wheelchair dependent.

### Survey

The online survey was developed with LimeSurvey® open-source survey tool version 3.25 (LimeSurvey GmbH, Hamburg, Germany), running on a HTTPS secured web server hosted by the University of Modena and Reggio Emilia. An individual token-based link to the survey was sent to all participants by e-mail. The questionnaire was composed of a few questions about sports or physical activities practiced at youth. As shown in Supplementary Fig. 1, the first question asked to consider only the sports or physical activities regularly organized in training sessions at least twice per week and for longer than 3 months consecutively during the period between 6 and 30 years of age. The questionnaire terminated if in this period no sports were practiced, and the answers were saved correctly. Conversely, in case of an affirmative answer, the person interviewed had to choose between one or more sports from a list, and for each sport was asked to indicate the age at which the sport began, and whether was ongoing at the time of the compilation or not. Moreover, the questionnaire asked to indicate whether their PA was considered “competitive” or rather organized into training sessions held on regularity or played at “amateur” level, without regular training sessions. Each person was linked to the personal identification number (ID) they were assigned upon registration in the INRF database. Information about gender, D4Z4 allele size, age at evaluation, age at onset, FSHD score, and clinical category were available at the INRF database and were connected to the survey’s answers. The survey has been open for answers between March and July 2021. Reading the attached informative sheet and the agreement with the informed consent were requested to participate in the survey.

### Outcome

The FSHD subcategory was considered as a stratification factor and all the associations of interest were assessed separately for each of the four FSHD subcategories, respectively category A (classical phenotype), category B (incomplete phenotype), category C (asymptomatic carrier), and category D (complex phenotype).

### Statistical analysis

The numerical variables were described as the mean ± standard deviation (SD), whereas the categorical variables as the absolute and percentage numbers. The association between PAs practiced between 6 and 30 years of age and the FSHD score was measured using the mean ratio (MR) with 95% confidence interval (CI), separately for each FSHD subcategory. Both unadjusted and confounding-adjusted MRs were calculated, by using Poisson regression models. The independent variables in the adjusted analysis were: PAs (yes vs. no); gender (M vs. F); disease duration (years); the size of DRA alleles (11–19 kb, 20–30 kb, 31–35 kb, 36–41 kb). The modification effect of PAs based on disease duration was assessed by significance testing of interaction terms. Statistical analyses were carried out by using R 3.6.3 software (The R Foundation for Statistical Computing, Wien) at a significance level equal to *p* < 0.05.

## Results

### Characteristics of participants

Descriptive characteristics of the enrolled individuals are reported in Table 1. The average age at the clinical evaluation of the FSHD score was 36.6 ± 9.4 years and 47.6% of them were male. The FSHD classical phenotype (subcategory A) was observed in 157 (42.7%) participants, whereas the incomplete phenotype with mild severity (category B) in 46 (12.5%) patients and the complex phenotype (category D) in 61 (16.6%) participants. Asymptomatic carriers were 104 (28.3%). The average (± SD) FSHD score was 5.8 ± 3.0 in participants with classical phenotype, 2.2 ± 1.7 in participants with incomplete phenotype with mild severity, 0.0 ± 0.2 in asymptomatic carriers, and 6.5 ± 3.8 in participants with a complex phenotype. The disease duration was longer for participants with a classical phenotype (15.8 ± 11.1 years) and complex phenotype (13.3 ± 11.9) than for participants with an incomplete phenotype (7.3 ± 9.0). Those who practiced PAs between 6 and 30 years of age for at least 3 consecutive months were 36.4% and most of them practiced organized PAs (26.9%) rather than non-organized PAs (9.5%). Moreover, most of them had practiced PAs with medium-high cardiovascular commitment (29.3%). Of those who practiced PAs during their lifetime, 134 (82.2%) exercised between 6 and 30 years of age for at least 3 consecutive months.

**Table 1:** Characteristics of patients

		Classical phenotype (n = 157)	Incomplete phenotype (n = 46)	Asymptomatic carrier (n = 104)	Complex phenotype (n = 61)
Age at FSHD score evaluation – years	mean ± SD	36.4 ± 9.0	35.6 ± 10.0	35.0 ± 9.5	40.5 ± 8.7
18–39	n (%)	88 (56.1%)	29 (63.0%)	66 (63.5%)	21 (34.4%)
40–60	n (%)	69 (43.9%)	17 (37.0%)	38 (36.5%)	40 (65.6%)
Age at symptoms onset – years (*)	mean ± SD	20.5 ± 10.4	28.3 ± 12.0	-	27.3 ± 14.3
0–17	n (%)	79 (50.3%)	12 (26.1%)	-	20 (32.8%)
18–39	n (%)	65 (41.4%)	26 (56.5%)	-	22 (36.1%)
40–60	n (%)	13 (8.3%)	8 (17.4%)	-	19 (31.1%)
Disease duration – years (*)	mean ± SD	15.8 ± 11.1	7.3 ± 9.0	-	13.3 ± 11.9
0–9	n (%)	56 (35.7%)	30 (65.2%)	-	30 (49.2%)
10–19	n (%)	47 (30.0%)	10 (21.7%)	-	12 (19.7%)
20–29	n (%)	33 (21.0%)	5 (10.9%)	-	13 (21.3%)
30–50	n (%)	21 (13.4%)	1 (2.2%)	-	6 (9.8%)
Gender - male	n (%)	81 (51.6%)	26 (56.5%)	39 (37.5%)	29 (47.5%)
Practiced Pas	n (%)	66 (42.0%)	24 (52.2%)	30 (28.8%)	14 (23.0%)
non-organized training	n (%)	17 (10.8%)	6 (13.0%)	7 (6.7%)	5 (8.2%)
organized training	n (%)	49 (31.2%)	18 (39.1%)	23 (22.1%)	9 (14.8%)
medium-high cardiovascular commitment	n (%)	51 (32.5%)	19 (41.3%)	26 (25.0%)	12 (19.7%)
FSHD score [0–15]	mean ± SD	5.8 ± 3.0	2.2 ± 1.7	0.0 ± 0.2	6.5 ± 3.8
0–1	n (%)	1 (0.6%)	21 (45.7%)	104 (100.0%)	5 (8.2%)
2–4	n (%)	62 (39.5%)	20 (43.5%)	0 (0.0%)	17 (27.9%)
5–10	n (%)	80 (51.0%)	5 (10.9%)	0 (0.0%)	28 (45.9%)
11–15	n (%)	14 (8.9%)	0 (0.0%)	0 (0.0%)	11 (18.0%)
DRA dimension – number of alleles	mean ± SD	24.7 ± 6.3	29.3 ± 5.3	32.7 ± 5.1	26.6 ± 7.1
11–19	n (%)	32 (20.4%)	2 (4.3%)	0 (0.0%)	9 (14.8%)
20–30	n (%)	97 (61.8%)	27 (58.7%)	33 (31.7%)	35 (57.4%)
31–35	n (%)	23 (14.6%)	10 (21.7%)	44 (42.3%)	13 (21.3%)
36–41	n (%)	5 (3.2%)	7 (15.2%)	27 (26.0%)	4 (6.6%)

Notes: PA = physical activity; DRA = D4Z4 reduced allele; SD = standard deviation

### Enrolment

Until December 2020 the INRF collected 3396 individuals with heterozygous D4Z4 Reduced alleles (DRA). The subjects who fulfilled the inclusion and exclusion criteria were 629, of which 424 (67.4%) had practiced PAs during their lifetime whereas 205 (32.6%) had not. The latter were included in the analysis, while 297 (70.0%) of the former were invited to participate to the online survey. People who were not invited to participate to the survey were those not aged between 18 and 60 years or whose e-mail address was not available in the INRF database. Subjects who attended the online survey and were included in the analysis were 163 (54.9% of those invited to participate). The total number of subjects included in the analysis was 368.

### Association of physical activity and FSHD score

As a primary aim, we evaluated 368 carriers of the DRA included in the study to assess whether there was an association between PAs practiced between 6 and 30 years of age and the FSHD score. The participants were clinically subdivided into different classes of phenotype in accordance with the CCEF. After adjusting for gender, disease duration and DRA size, we observed that the FSHD score was lower in the group who had practiced PAs in comparison with the group who did not. This difference is estimated to be 17% in participants with the classical phenotype (MR = 0.83, 95% CI = 0.73–0.95, *p* = 0.007) and 33% in participants with the complex phenotype (MR = 0.67, 95% CI = 0.51–0.89, *p* = 0.006). This difference is statistically significant (Table 2). Among the DRA carriers a notable number of individuals practiced organized PAs, most of them with medium-high cardiovascular commitment. Hence, as a secondary aim we investigated if there was an analogue association between the practice of organized or medium-high cardiovascular commitment physical activities and FSHD severity. We observed again a reduction of 15% and 13% in FSHD score, in participants with classical phenotype (MR = 0.85, 95% CI = 0.74–0.99, *p* = 0.031 and MR = 0.87, 95% CI = 0.75–1.01, *p* = 0.061) and of 42% and 39% reduction in participants with complex phenotype (MR = 0.58, 95% CI = 0.41–0.83, *p* = 0.002 and MR = 0.61, 95% CI = 0.45–0.83, *p* = 0.002), respectively (Table 3). Conversely, no difference was observed in participants with incomplete phenotype with mild severity either if any PA (MR = 0.87, 95% CI = 0.56–1.34, *p* = 0.524) or organized PA (MR = 0.83, 95% CI = 0.55–1.26, *p* = 0.374) or PA with medium-high cardiovascular commitment (MR = 0.99, 95% CI = 0.64–1.52, *p* = 0.952) was practiced (Table 1). The tendency observed in subcategories A and D of the group that practiced exercise seems to be constant over the disease course, as there was no effect modification related to disease duration and either any PA (*p* = 0.730 and *p* = 0.287 for subcategories A and D, respectively), organized PA (*p* = 0.180 and *p* = 0.409) or PA with medium-high cardiovascular commitment (*p* = 0.851 and *p* = 0.671). The comparisons of the FSHD score between DRA carriers who practiced PA and did not, showed a low statistical power at the *post hoc* calculation, ranging from 0.10 (Category B) to 0.58 (Category A and D). As FSHD score was zero in Category C the calculation was not performed.

**Table 2:** Association of physical activities and FSHD severity

Subcategory	FSHD score		MR (95% CI)	*p*-value	aMR (95% CI)	*p*-value
	n	mean ± SD				
**Classical phenotype**						
No PAs	91	6.29 ± 3.01	Reference	-	Reference	-
PAs	66	5.24 ± 2.90	0.83 (0.73; 0.95)	0.008 *	0.83 (0.73; 0.95)	0.007 *
**Incomplete phenotype**						
No PAs	22	2.00 ± 1.63	Reference	-	Reference	-
PAs	24	2.33 ± 1.76	1.17 (0.79; 1.73)	0.444	0.87 (0.56; 1.34)	0.524
**Asymptomatic carriers**						
No PAs	74	0.00 ± 0.00	Reference	-	Reference	-
PAs	30	0.10 ± 0.31	NA	NA	NA	NA
**Complex phenotype**						
No PAs	47	7.06 ± 3.87	Reference	-	Reference	-
PAs	14	4.71 ± 3.27	0.67 (0.51; 0.87)	0.003 *	0.67 (0.51; 0.89)	0.006 *

Notes. PA = physical activity; SD = standard deviation; MR = mean ratio; aMR = MR adjusted for gender, disease duration and DRA dimension; CI = confidence interval; * = *p*-value < 0.05

**Table 3:** Association of organized or at medium-high cardiovascular commitment physical activities and FSHD severity

Subcategory	FSHD score		MR (95% CI)	*p*-value	aMR (95% CI)	*p*-value
	n	mean ± SD				
**Organized PAs**						
**Classical phenotype**						
No organized PAs	108	6.13 ± 3.09	Reference	-	Reference	-
Organized Pas	49	5.20 ± 2.73	0.85 (0.73; 0.98)	0.025 *	0.85 (0.74; 0.99)	0.031 *
**Incomplete phenotype**						
No organized PAs	28	2.14 ± 1.88	Reference	-	Reference	-
Organized Pas	18	2.22 ± 1.40	1.04 (0.70; 1.55)	0.859	0.83 (0.55; 1.26)	0.374
**Asymptomatic carriers**						
No organized PAs	81	0.00 ± 0.00	Reference	-	Reference	-
Organized Pas	23	0.13 ± 0.34	NA	NA	NA	NA
**Complex phenotype**						
No organized PAs	52	6.98 ± 3.77	Reference	-	Reference	-
Organized Pas	9	3.89 ± 3.30	0.56 (0.39; 0.79)	0.001 *	0.58 (0.41; 0.83)	0.002 *
**Medium-high cardiovascular commitment PAs**						
**Classical phenotype**						
No MHCC Pas	106	6.17 ± 3.08	Reference	-	Reference	-
MHCC Pas	51	5.18 ± 2.75	0.84 (0.73; 0.97)	0.016 *	0.87 (0.75; 1.01)	0.061
**Incomplete phenotype**						
No MHCC Pas	27	1.93 ± 1.52	Reference	-	Reference	-
MHCC Pas	19	2.53 ± 1.90	1.31 (0.89; 1.94)	0.175	0.99 (0.64; 1.52)	0.952
**Asymptomatic carriers**						
No MHCC Pas	78	0.00 ± 0.00	Reference	-	Reference	-
MHCC Pas	26	0.12 ± 0.33	NA	NA	NA	NA
**Complex phenotype**						
No MHCC Pas	49	7.10 ± 3.84	Reference	-	Reference	-
MHCC Pas	12	4.17 ± 2.95	0.59 (0.44; 0.79)	0.000 *	0.61 (0.45; 0.83)	0.002 *

Notes: MHCC = medium-high cardiovascular commitment; SD = standard deviation; MR = mean ratio; aMR = MR adjusted for gender, disease duration and DRA dimension; CI = confidence interval; * = *p*-value < 0.05

## Discussion

The major finding of the present work is that, in FSHD participants belonging to A and D clinical categories, PAs performed at a young age are associated with a lower clinical score in the subsequent stages of the disease.

It is well known that PAs and subsequent energy expenditure, are strictly associated with a longer lifespan and maintenance/amelioration of muscular structure and function not only in healthy people, but also in those with chronic and deadly age-related diseases [20]. However, to the best of our knowledge, this is the first study in which the association of previously practiced PAs and the subsequent long-term clinical disease manifestation in FSHD patients is investigated. The association between disease severity and PAs practiced at young age in clinically stratified participants carrying D4Z4 reduced allele was retrospectively analyzed. The results show that, in our sample, the practice of organized activities for at least 3 months consecutively is associated with lower clinical score. This association was stronger in those presenting FSHD classic or complex phenotypes who practiced PAs with medium-high cardiovascular commitment. No association was found in the group presenting incomplete phenotype with mild severity (category B). One possible explanation is that participants in the latter subcategory usually do not show severe muscle deficits to make such a difference detectable. It is also possible that the lower sample size of the subcategory B might have affected the precision of that association. Overall, results from our study seem to indicate that PAs performed between 6 and 30 years may positively impact on the subsequent clinical picture of the disease and may represent useful information on the correct lifestyles to be adopted by the DRA asymptomatic carriers. These results are consistent with what has been reported in the literature regarding the effect of PAs in FSHD. Indeed, several positive effects have been prospectively attributed to PAs in FSHD as outlined by three recently published randomized controlled trials (RCTs). These studies showed ameliorative effects due to endurance training, High-Intensity Interval Training (HIIT), and possibly, to moderate strength training on the aerobic capacity, walking speed, muscular strength and fatigue [21–23]. The retrospective nature of the study does not allow to draw firm conclusions regarding the physiological mechanisms underlying the present findings. However, we can speculate that PA may lead to a better skeletal muscle substrate [24, 25] on which the degenerative process subsequently acts. Additional implications of the obtained results may be drawn in the frame of clinical research. In particular, the systematic and standardized collection of a large amount of data is crucial for the understanding of a rare complex disease such as FSHD. From this point of view the INRF, which integrates anamnestic records with clinical and molecular features, provides a realistic observation of the phenotypic variability and can provide hints on other risk factors that can contribute to amelioration or worsening of the disease, such as PAs.

### Limitations

This study has some limitations: due to the retrospective design of the study, we cannot completely rule out the risk of bias related to unmeasured confounders, selection of patients or data collection. For example, we cannot exclude flaws, particularly in the older patients, due to inaccurate memory of the physical activity carried out in the range of age taken into account. Nevertheless, such a design allowed to study the long-term effect of PAs practiced at young age and to assess their role on FSHD presentation in adulthood. Furthermore, it cannot be overlooked that the association observed here could be influenced by the fact that participants with better physical function may have been more incline to maintain a higher level of exercise compared to participants who experienced more severe physical limitations. Similarly, we are not able to ascertain whether, within the control group, the declared lack of exercise was dictated by subtle manifestations of the disease which made the subjects less incline to exercise’s practice. Certainly, future longitudinal studies on exercise’s effects in DRA carriers will be necessary to confirm or deny what has been reported.

## Conclusions

Overall, our study shows that the practice of PAs at young age is not detrimental in DRA carriers regardless of the clinical category and suggests a positive association, unlike causative, on the subsequent clinical score in A and D subcategory. This underlines the importance of investigating previously practiced PAs to obtain insights on the effects of factors influencing disease progression. Future prospective controlled studies will be needed to unravel the mechanisms underlying the lower clinical severity in FSHD devoted to PAs and to understand whether PAs practiced at young age can delay disease onset.

### Electronic supplementary material

Below is the link to the electronic supplementary material.

Supplemental Table 1

Supplemental Table 2

Supplementary Fig. 1

## Data Availability

The datasets used and analysed during the current study are available from the corresponding author on reasonable request.

## Abbreviations

FSHD: facioscapulohumeral muscular dystrophy
PA: physical activity
DRA: D4Z4 reduced allele
CCEF: Comprehensive Clinical Evaluation Form
NMD: neuromuscular disease
BMI: body mass index
INRF: Italian National Registry for FSHD
HR: Heart Rate
CO: Cardiac Output
PR: Peripheral Resistance
LCC: Low Cardiovascular Commitment
MCC: Moderate Cardiovascular Commitment
MHCC: Moderate-High Cardiovascular Commitment
ID: identification number
MR: mean ratio
CI: confidence interval
RCT: randomized controlled trial
HIIT: High-Intensity Interval Training
