# Supplementary material for: Physical activity practiced at a young age is associated with a less severe subsequent clinical presentation in facioscapulohumeral muscular dystrophy
Source: BMC Musculoskelet Disord. 2024 Jan 5;25:35. doi: 10.1186/s12891-023-07150-x (PMC10768364; doi:10.1186/s12891-023-07150-x)
Supplement: Supplementary file 1 — Supplemental Table 1 [file 12891_2023_7150_MOESM1_ESM.docx]

| **Table S1. Classification of sports based on cardiovascular commitment** | | | |
| --- | --- | --- | --- |
|  | HR | PR | CO |
| Group A | + | = | =/+ |
| Group B | ++/+++ | =/+ | =/+ |
| Group C | ++/+++ | ++/+++ | ++ |
| Group D1 | +/++/+++ | +/++ | +/++ |
| Group D2 | ++/+++ | -/= | ++/+++ |

**Table S1** shows the evaluation criteria based on Heart Rate (HR), Peripheral Resistance (PR) and Cardiac Output (CO), that permitted to classify sports into 5 different groups. Groups A and B include sport with low cardiovascular commitment (LCC), group C includes sport with moderate cardiovascular commitment (MCC) and finally groups D1 and D2 involve sport with medium-high cardiovascular commitment (MHCC). Modified by COCIS 2009.
