# Supplementary material for: Physical activity practiced at a young age is associated with a less severe subsequent clinical presentation in facioscapulohumeral muscular dystrophy
Source: BMC Musculoskelet Disord. 2024 Jan 5;25:35. doi: 10.1186/s12891-023-07150-x (PMC10768364; doi:10.1186/s12891-023-07150-x)
Supplement: Supplementary file 2 — Supplemental Table 2 [file 12891_2023_7150_MOESM2_ESM.docx]

**Table S2. Sports classification related to the enrolled patients according to COCIS 2009 report**.

|  |  |
| --- | --- |
| Motoring | GROUP B |
| American football | GROUP D1 |
| Shot put, hammer throw, javelin throw, discus throw | GROUP C |
| Hockey | GROUP D1 |
| Handball | GROUP D1 |
| Rugby | GROUP D1 |
| Fencing | GROUP D1 |
| Baseball | GROUP D1 |
| Football (7 players) | GROUP D1 |
| Track, road cycling | GROUP D2 |
| Hydrobike | GROUP A |
| Water polo | GROUP D1 |
| Ultimate fresbee | GROUP D1 |
| Rowing, kayaking | GROUP D1 |
| Marathon (half/full marathon, ultramarathon), march | GROUP E |
| Motocross | GROUP C |
| Table tennis | GROUP C |
| Scuba-diving | GROUP B |
| High jump, long jump, triple jump | GROUP C |
| Sailing | GROUP B |
| Skating, Ice Skating | GROUP D1 |
| Yoga | GROUP A |
| Water aerobics | GROUP A |
| Track running (speed, middle distance, obstacles, relay race) | GROUP D2 |
| Rhythmic Gymnastics | GROUP D1 |
| Jogging | GROUP A |
| Tennis | GROUP D1 |
| Bodybuilding | GROUP C |
| Martial art | GROUP D1 |
| Basketball | GROUP D1 |
| Skiing | GROUP C |
| Hiking, Nordic Walking, Trekking | GROUP A |
| Dance | GROUP B |
| Cycle | GROUP A |
| Volleyball/Beach volleyball | GROUP D1 |
| Football | GROUP D1 |
| Gym (Aerobics Classes, Pilates, Etc.) | GROUP D1 |
| Swimming | GROUP A |
| Riding | GROUP B |
| Bowls | GROUP B |
| Archery | GROUP A |
| Go-Karting | GROUP B |
| Boxe | GROUP D1 |
