# Supplementary material for: Physical activity practiced at a young age is associated with a less severe subsequent clinical presentation in facioscapulohumeral muscular dystrophy
Source: BMC Musculoskelet Disord. 2024 Jan 5;25:35. doi: 10.1186/s12891-023-07150-x (PMC10768364; doi:10.1186/s12891-023-07150-x)
Supplement: Supplementary file 3 — Supplementary Fig. 1 [file 12891_2023_7150_MOESM3_ESM.pdf]

Protocol N. 0016153/18

Subject unique ID |_|_|_|_|/|_|_| To be completed by the Investigators

Sex
- O Male
- O Female

Did you practice any sports or physical activities for at least 3 consecutive months and with training sessions twice a week (at least) when you were aged between 6 and 30 years old?
- O No
- O Yes

If yes, please provide

Sport	Practiced	Level of practice	Starting age	Ongoing?
Water aerobics	○ No<br>○ Yes	○ Amateur/recreational<br>○ Competitive	|_|_| years	○ No<br>○ Yes
Martial arts	○ No<br>○ Yes	○ Amateur/recreational<br>○ Competitive	|_|_| years	○ No<br>○ Yes
Motoring	○ No<br>○ Yes	○ Amateur/recreational<br>○ Competitive	|_|_| years	○ No<br>○ Yes
Dance	○ No<br>○ Yes	○ Amateur/recreational<br>○ Competitive	|_|_| years	○ No<br>○ Yes
Baseball	○ No<br>○ Yes	○ Amateur/recreational<br>○ Competitive	|_|_| years	○ No<br>○ Yes
Cycle	○ No<br>○ Yes	○ Amateur/recreational<br>○ Competitive	|_|_| years	○ No<br>○ Yes
Foosball	○ No<br>○ Yes	○ Amateur/recreational<br>○ Competitive	|_|_| years	○ No<br>○ Yes
Soccer (7 players)	○ No<br>○ Yes	○ Amateur/recreational<br>○ Competitive	|_|_| years	○ No<br>○ Yes
Hiking, Nordic Walking, Trekking	○ No<br>○ Yes	○ Amateur/recreational<br>○ Competitive	|_|_| years	○ No<br>○ Yes
Rowing, Kayaking	○ No<br>○ Yes	○ Amateur/recreational<br>○ Competitive	|_|_| years	○ No<br>○ Yes
Track/Road cycling	○ No<br>○ Yes	○ Amateur/recreational<br>○ Competitive	|_|_| years	○ No<br>○ Yes
Track Running (speed, middle distance, obstacle, relay race)	○ No<br>○ Yes	○ Amateur/recreational<br>○ Competitive	|_|_| years	○ No<br>○ Yes
American football	○ No<br>○ Yes	○ Amateur/recreational<br>○ Competitive	|_|_| years	○ No<br>○ Yes
Shot put, hammer throw, javelin throw, discus throw	○ No<br>○ Yes	○ Amateur/recreational<br>○ Competitive	|_|_| years	○ No<br>○ Yes
Hockey	○ No<br>○ Yes	○ Amateur/recreational<br>○ Competitive	|_|_| years	○ No<br>○ Yes
Rhythmic gymnastics	○ No<br>○ Yes	○ Amateur/recreational<br>○ Competitive	|_|_| years	○ No<br>○ Yes
Hydrobike	○ No<br>○ Yes	○ Amateur/recreational<br>○ Competitive	|_|_| years	○ No<br>○ Yes
Jogging	○ No<br>○ Yes	○ Amateur/recreational<br>○ Competitive	|_|_| years	○ No<br>○ Yes
Marathon (half/full-marathon, ultra-marathon), march	○ No<br>○ Yes	○ Amateur/recreational<br>○ Competitive	|_|_| years	○ No<br>○ Yes
Motocross	○ No<br>○ Yes	○ Amateur/recreational<br>○ Competitive	|_|_| years	○ No<br>○ Yes
Swimming	○ No<br>○ Yes	○ Amateur/recreational<br>○ Competitive	|_|_| years	○ No<br>○ Yes
Gymnastics (aerobics, pilates, etc…)	○ No<br>○ Yes	○ Amateur/recreational<br>○ Competitive	|_|_| years	○ No<br>○ Yes

Sport	Practiced	Level of practice	Starting age	Ongoing?
Basketball	○ No<br>○ Yes	○ Amateur/recreational<br>○ Competitive	|_|_| years	○ No<br>○ Yes
Handball	○ No<br>○ Yes	○ Amateur/recreational<br>○ Competitive	|_|_| years	○ No<br>○ Yes
Water polo	○ No<br>○ Yes	○ Amateur/recreational<br>○ Competitive	|_|_| years	○ No<br>○ Yes
Volleyball/Beach Volley	○ No<br>○ Yes	○ Amateur/recreational<br>○ Competitive	|_|_| years	○ No<br>○ Yes
Skating/Ice skating	○ No<br>○ Yes	○ Amateur/recreational<br>○ Competitive	|_|_| years	○ No<br>○ Yes
Pentathlon	○ No<br>○ Yes	○ Amateur/recreational<br>○ Competitive	|_|_| years	○ No<br>○ Yes
Weightlifting/Body building	○ No<br>○ Yes	○ Amateur/recreational<br>○ Competitive	|_|_| years	○ No<br>○ Yes
Table tennis	○ No<br>○ Yes	○ Amateur/recreational<br>○ Competitive	|_|_| years	○ No<br>○ Yes
Rugby	○ No<br>○ Yes	○ Amateur/recreational<br>○ Competitive	|_|_| years	○ No<br>○ Yes
High jump, long jump, triple jump	○ No<br>○ Yes	○ Amateur/recreational<br>○ Competitive	|_|_| years	○ No<br>○ Yes
Pole-vault	○ No<br>○ Yes	○ Amateur/recreational<br>○ Competitive	|_|_| years	○ No<br>○ Yes
Fencing	○ No<br>○ Yes	○ Amateur/recreational<br>○ Competitive	|_|_| years	○ No<br>○ Yes
Skiing	○ No<br>○ Yes	○ Amateur/recreational<br>○ Competitive	|_|_| years	○ No<br>○ Yes
Water skiing	○ No<br>○ Yes	○ Amateur/recreational<br>○ Competitive	|_|_| years	○ No<br>○ Yes
Scuba-diving	○ No<br>○ Yes	○ Amateur/recreational<br>○ Competitive	|_|_| years	○ No<br>○ Yes
Ultimate frisbee	○ No<br>○ Yes	○ Amateur/recreational<br>○ Competitive	|_|_| years	○ No<br>○ Yes
Tennis	○ No<br>○ Yes	○ Amateur/recreational<br>○ Competitive	|_|_| years	○ No<br>○ Yes
Triathlon	○ No<br>○ Yes	○ Amateur/recreational<br>○ Competitive	|_|_| years	○ No<br>○ Yes
Twirling	○ No<br>○ Yes	○ Amateur/recreational<br>○ Competitive	|_|_| years	○ No<br>○ Yes
Sailing	○ No<br>○ Yes	○ Amateur/recreational<br>○ Competitive	|_|_| years	○ No<br>○ Yes
Yoga	○ No<br>○ Yes	○ Amateur/recreational<br>○ Competitive	|_|_| years	○ No<br>○ Yes
Other, please specify<br>________________________	○ No<br>○ Yes	○ Amateur/recreational<br>○ Competitive	|_|_| years	○ No<br>○ Yes

Supplementary Figure 1: Questionnaire
